# Secondary syphilis presenting with non-arteritic anterior ischemic optic neuropathy (NAION)

**DOI:** 10.1093/omcr/omae012

**Published:** 2024-03-25

**Authors:** Fares A AlKhayal, Moath K Albusair

**Affiliations:** Dermatology and Dermatologic Surgery Department, Prince Sultan Military Medical City, Riyadh, Saudi Arabia; Dermatology and Dermatologic Surgery Department, Prince Sultan Military Medical City, Riyadh, Saudi Arabia

**Keywords:** secondary syphilis, NAION, neurosyphilis

## Abstract

Syphilis is a sexually transmitted disease caused by the spirochete bacterium *Treponema pallidum.* Syphilis is a significant public health issue, notably in (HIV) positive patients. Due to the absence of pathognomonic signs in secondary syphilis and its ability to present and mimic a wide variety of clinical findings, it gained the name “the Great imitator ‘(mimicker).’ Herein, we describe a case of a 51-year-old man who presented with acute painless loss of vision of the right eye preceded by a few erythematous plaques with thick scales over bilateral legs and multiple discrete and confluent scaly papules over the palms and soles. During the hospital stay, a diagnosis of non-arteritic anterior ischemic optic neuropathy (NAION) as a manifestation of neurosyphilis is made. To the best of our knowledge, this is the first reported case of NAION as the presenting symptom of neurosyphilis in secondary syphilis in an immunocompetent patient.

## INTRODUCTION

*Treponema pallidum*, a gram-negative spirochete, is the causative organism of sexually transmitted disease syphilis [1]. An estimated 6 million new cases of syphilis are reported annually worldwide, with developing nations accounting for the majority of cases [2]. Syphilis is a serious public health concern, and as high-risk sexual conduct has become more common, so has the frequency of Syphilitic infections, especially in those who are HIV positive [3]. Retrospective research revealed that 49.3% of syphilitic cases are HIV positive [4]. Three primary stages have been identified for these presentations. The incubation period for primary syphilis is 10 to 90 days following infection [5]. Small papules typically start and develop into a distinct, painless, indurated red-based ulcer (chancre) at the location of infection with lymph node enlargement [6]. secondary syphilis is a result of hematogenous and lymphatic infection spreading. It occurs a few weeks or months post chancre, with various possible systemic symptoms and cutaneous manifestations, including a general body rash (often including the palmar and plantar surfaces), alopecia, and others [7, 8]. The absence of pathognomonic signs, and the ability to present in a wide range of clinical presentations and to mimic any systemic inflammatory diseases, is often referred to as the ‘the Great imitator’ [1]. It is frequently referred to as ‘the Great imitator’ due to its lack of pathognomonic signs, versatility in clinical presentation, and capacity to imitate any systemic inflammatory disease [1]. Tertiary latent syphilis is an asymptomatic phase that can only be identified by serologic testing. Early latent syphilis is defined as an infection that happened within the previous 12 months and has the potential to relapse into the secondary stage. After that, syphilis is categorized as late latent, meaning that sexual transmission is improbable if the illness manifested more than a year ago. However, tertiary syphilis can still occur and present as neurological, cardiovascular, gummous manifestations.

## CASE REPORT

**Figure 1:**
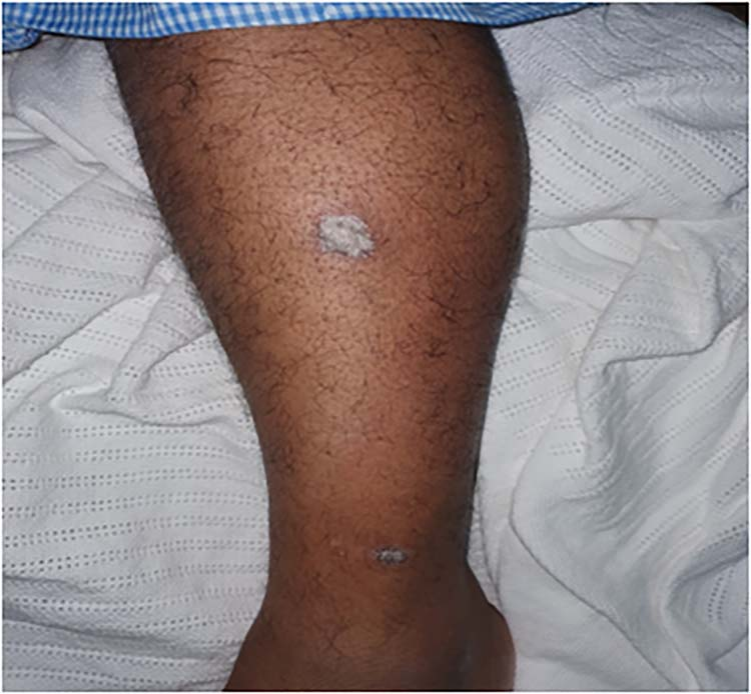
Well-defined erythematous thick scaly plaques over the lower limbs.

**Figure 2:**
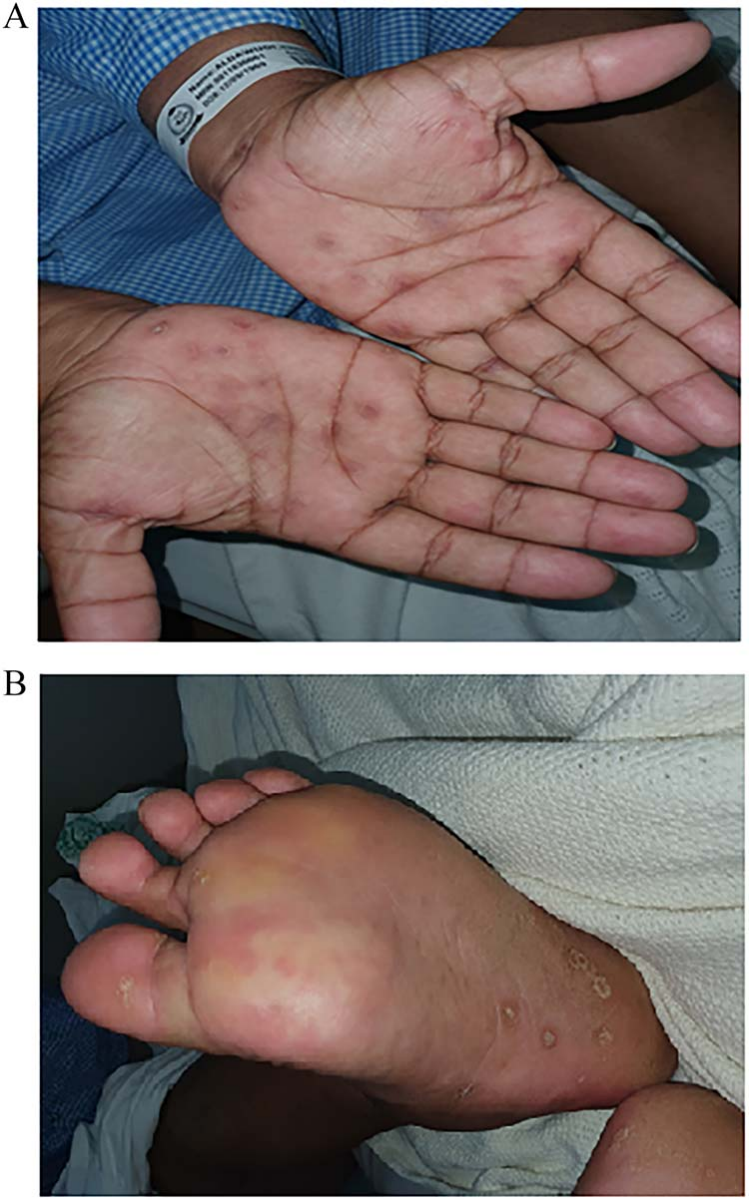
(**A**) Multiple discrete and confluent scaly papules over the palms. (**B**) Multiple discrete and confluent scaly papules over the soles.

**Figure 3:**
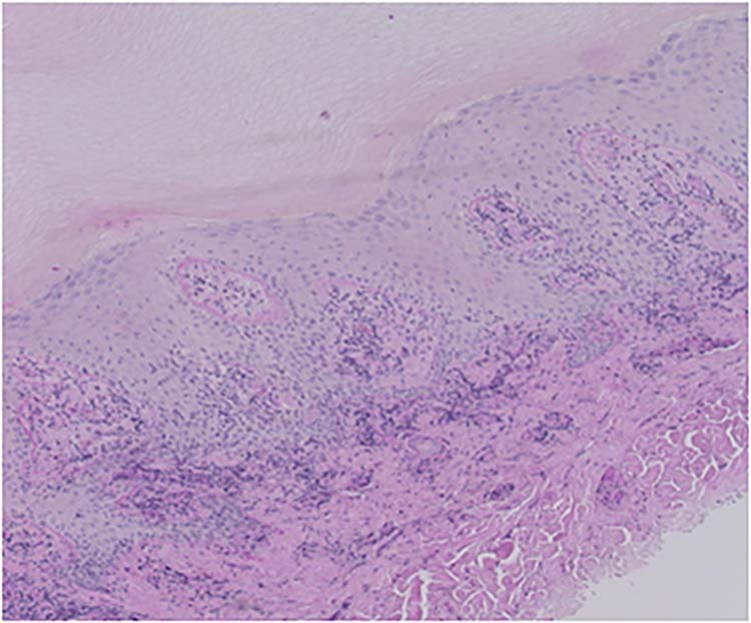
Section Showed hyper granulosis with band-like inflammation and saw tooth rete ridges. The dermal inflammatory cell infiltrate is partially crushed; hence, presence of plasma cells couldn’t be confirmed. There is no evidence of psoriasis.

A 51-year-old male known to have DM and HTN presented to the ER department with a complaint of acute painless loss of vision over the right eye. An ophthalmologic assessment revealed non-arteritic anterior ischemic optic neuropathy (NAION). Furthermore, the patient was referred to dermatology to evaluate skin rash over the lower leg. The skin examination showed a few erythematous plaques with thick scales over bilateral legs (Fig. 1). In addition, multiple discrete and confluent scaly papules were over the palms and soles. (Fig. 2A and B). The skin biopsy, which was taken from the right palm, showed hypergranulosis with band-like inflammation and saw tooth ridges. The dermal inflammatory cell infiltrate is partially crushed; hence, the presence of plasma cells cannot be confirmed. Warthin Starry stain wasn’t available at that time (Fig. 3), treponemal (IgM Antibody—FTA-ABS), and nontreponemal (IgM & IgG—VDRL) were positive. Cerebrospinal fluid (CSF) examination showed High levels of protein and glucose with WBC count>136 cumm with a negative culture of other bacterial and viral infections. Neisseria gonorrhea and chlamydia test were negative. The patient was tested also negative for HIV. Hence Diagnosis of secondary syphilis with neurosyphilis manifestation is made, and the patient received Ceftriaxone, 2 g IM daily for ten days with calcipotriol ointment, with total clearance of patient skin rash.

## DISCUSSION

Secondary syphilis lesions occur anywhere on the body, including palms and soles, with polymorphic myriad presentations most commonly in the form of generalized, non-pruritic papulosquamous copper-colored rash involving the palms and soles (80%) accompanied by prodromal signs such as low-grade fever, arthralgia, headache, fatigue, malaise, and sore throat [7]. Other manifestations include condylomata lata, hypopigmented macules on the neck (also known as the ‘necklace of Venus’), lichenoid rash, and ‘moth-eaten’ baldness [8]. Therefore, when examining any skin rash of unknown etiology, it should be considered in the differential diagnosis, particularly in sexually active patients. Ocular syphilis was 3% common in patients with secondary syphilis in the pre-antibiotic era. Ocular involvement in syphilis has been less common since the discovery of antibiotics, but it is still a significant symptom to be aware of because ocular symptoms of syphilis are frequently misunderstood because they lack conventional features [9].

Ocular syphilitic infection occurs mainly in secondary and tertiary syphilis [10]. It may be present as silent or can virtually affect any components of the eyes, uveitis being the most common form. In this report, others include retinitis, optic neuritis, choroiditis, interstitial keratitis, retinal vasculitis, and non-arteritic anterior ischemic optic neuropathy (NAION) [11].

Ocular syphilis has been described in both immunocompetent and immunocompromised patients [9]. Thus, it is recommended to test for HIV in individuals who present with ocular syphilis. However, It is still controversial whether ocular involvement of syphilis is synonymous with neurosyphilis and, thus, the relevance of performing LP [9]. Non-arteritic anterior ischemic optic neuropathy (NAION) is the most common acute optic neuropathy in patients over 50 years of age with vasculopathy risk, and it’s a clinical diagnosis. Characteristics features in eye funds exams include optic disk edema, splinter hemorrhage, or dilated capillaries [12]. Following glaucoma, it is the second most common cause of permanent optic nerve-related visual loss in adults [13].

Patients typically present with acute, painless, unilateral loss of vision, most reported upon awakening with no clear pathogenesis [14].

In most patients diagnosed with NAION, Visual acuity usually remains the same or improves slightly. However, in a small number of patients, visual acuity may worsen in the first few weeks (progressive NAION) [15].

## CONCLUSION

The non-pruritic papulosquamous eruption is the most observed clinical presentation in secondary syphilis. Although neurosyphilis classically is a manifestation of tertiary syphilis, neurosyphilis can occur at any stage of the disease. Ocular syphilitic infection occurs mainly in secondary and tertiary syphilis. It may be present as silent or can virtually affect any components of the eyes, uveitis being the most common form.

## CONFLICT OF INTEREST STATEMENT

There is no conflict of interest to disclose.

## FUNDING

None.

## ETHICAL APPROVAL

Patient information was de-identified and consent for publication has been obtained.

## CONSENT

This case study has been published with the written consent of the patient involved.

## GUARANTOR

Fares alkhayal.
